# Nomogram Based on microRNA Signature Contributes to Improve Survival Prediction of Clear Cell Renal Cell Carcinoma

**DOI:** 10.1155/2020/7434737

**Published:** 2020-03-24

**Authors:** Enfa Zhao, Xiaofang Bai

**Affiliations:** ^1^Department of Structural Heart Disease, The First Affiliated Hospital of Xi'an Jiaotong University, Xi'an 710061, China; ^2^Department of Ultrasound Medicine, The First Affiliated Hospital of Xi'an Jiaotong University, Xi'an 710061, China

## Abstract

**Objective:**

Numerous microRNAs (miRNAs) have been identified in ccRCC and recommended to be used for predicting clear cell renal cell carcinoma (ccRCC) prognosis. However, it is not clear whether a miRNA-based nomogram results in improved survival prediction in patients with ccRCC.

**Methods:**

miRNA profiles from tumors and normal tissues were downloaded from The Cancer Genome Atlas (TCGA) database and analyzed using the “limma” package. The association between differentially expressed miRNAs and patient prognosis was identified using univariate, least absolute shrinkage and selection operator (LASSO), and multivariate Cox regression analyses. Next, all patients were randomly divided into development and validation cohorts at a ratio of 1 : 1. A nomogram was established based on independent prognostic factors in the development cohort. The prognostic performance of the nomogram was validated in both cohorts using the concordance index (C-index) and calibration plots.

**Results:**

Multivariate Cox analysis identified the 13-miRNA signature, as well as AJCC stage and age, as independent prognostic factors after adjusting for other clinical covariates. The nomogram was built based on the independent variables. In the development cohort, the C-index for the constructed nomogram to predict overall survival (OS) was 0.792, which was higher than the C-index (0.731) of the AJCC staging system and C-index (0.778) of the miRNA signature. The nomogram demonstrated good discriminative ability in the validation cohort in predicting OS, with a C-index of 0.762. The calibration plots indicated an excellent agreement between the nomogram predicted survival probability and the actual observed outcomes. Furthermore, decision curve analysis (DCA) indicated that the nomogram was superior to the AJCC staging system in increasing the net clinical benefit.

**Conclusions:**

The novel proposed nomogram based on a miRNA signature is a more reliable and robust tool for predicting the OS of patients with ccRCC compared to AJCC staging system, thus, improving clinical decision-making.

## 1. Introduction

Renal cell carcinoma (RCC) is a common genitourinary malignant tumor, accounting for 2–3% of all adult cancers, resulting in over 100,000 annual deaths worldwide [1, 2]. More than 66,800 newly diagnosed renal cancer patients and 13,860 deaths were reported in China in 2014 [3]. In the USA, approximately 63,920 newly diagnosed individuals and 13,860 renal cancer-related deaths were reported in 2014 [4]. The most common subtype of renal cancer is ccRCC, which accounts for 70–80% of all RCC cases [5].

microRNAs (miRNAs) are small noncoding RNAs consisting of 19–25 nucleotides that function in the regulation of protein-coding or noncoding gene expression, mainly by binding to the 3′untranslated region of target mRNAs [6]. Recently, many studies have revealed that abnormal miRNA expression can be utilized to evaluate the clinical prognosis of patients with various cancers, including ccRCC [7–11]. Currently, the prognosis of renal cancer mainly relies on the American Joint Committee on Cancer (AJCC) TNM staging system [12, 13]. However, the efficiency of its clinical application in predicting prognosis remains controversial [14, 15]. Moreover, the system does not incorporate other vital prognostic variables, such as age, sex, tumor size, primary site, and differentiation. Therefore, the development of a novel prognostic nomogram incorporating miRNA would be advantageous. To our knowledge, the availability of prognostic nomogram incorporating a miRNA signature is limited for patients with ccRCC. This study aimed to identify a miRNA signature and formulate a predictive nomogram which incorporates independent prognostic factors. The performance of the nomogram was compared with the presently used AJCC staging system.

## 2. Materials and Methods

### 2.1. Patients and Differentially Expressed miRNAs

In this study, miRNA expression data and clinical information from TCGA data portal (http://tcga.cancer.gov) were used. After excluding patients with incomplete clinical pathological information, a total of 504 patients were included. The ccRCC miRNA expression data were derived from 616 specimens, including 545 renal cancer specimens and 71 nontumor tissues. The “edgeR” package in the R software was used to screen differentially expressed miRNAs between cancer and normal tissues with a threshold of ∣log2foldchange (log2FC)∣ > 1 and adjusted *P* value <0.05 [16].

### 2.2. Identification of a Potential Prognostic miRNAs Signature

Univariate Cox analyses were performed to identify the link between the expression level of miRNAs and patient survival, with statistical significance set at *P* < 0.05. LASSO analysis was then conducted on the miRNAs founded to be statistically significant in the univariate analysis. It conducted a subselection of miRNAs via shrinkage of the regression coefficient by way of forcing a penalty proportional to their size [17]. The miRNAs with a *P* value <0.05 were further put through a multivariate Cox regression analysis to determinate the predictive miRNAs. Subsequently, a prognostic miRNA signature was established according to a liner combination of the expression levels of prognostic miRNAs weighted by the evaluated regression coefficient in the multivariate Cox regression model.

### 2.3. Identification of Independent Prognostic Variables and Nomogram Construction

The miRNA signature and other clinical variables were merged. The 504 patients were randomly divided into development (*n* = 252) and validation (*n* = 252) cohorts. To identify independent prognostic variables related to patient survival in the development cohort, univariate and multivariate Cox regression analysis were performed using prognosis as the dependent variable and miRNA signature and other related clinical factors including age, gender, stage, grade, and laterality as independent variables. Nomogram for the predictions of 1-, 3-, and 5-year survival was conducted according to the independent variables in the development cohort. The nomogram and calibration plots were performed using the R software (version 3.5.1, http://www.r-project.org/) with “rms” package.

### 2.4. Calibration and Validation of the Nomogram

The performance of our nomogram was validated by measuring the concordance index (C-index) and calibration curves in both cohorts [18]. Calibration plots were applied to examine the performance of the nomogram. A calibration plot along the 45-degree line in two cohorts would present an excellent calibration model between the bootstrap-predicted probabilities and the actual outcomes. The nomogram and AJCC staging system were compared using the rcorrp.cens package in R. The decision curve analysis (DCA) is a novel method that evaluates predictive models from the perspective of clinical consequences. DCA was performed in the entire cohort to test the clinical usefulness of the nomogram in comparison with the present AJCC staging system [19]. All statistical analyses were performed using the R software (v3.5.1, http://www.r-project.org). A two-tailed *P* value <0.05 was considered statistically significant.

## 3. Results

### 3.1. Identification of the miRNA Signature

**Figure 1:**
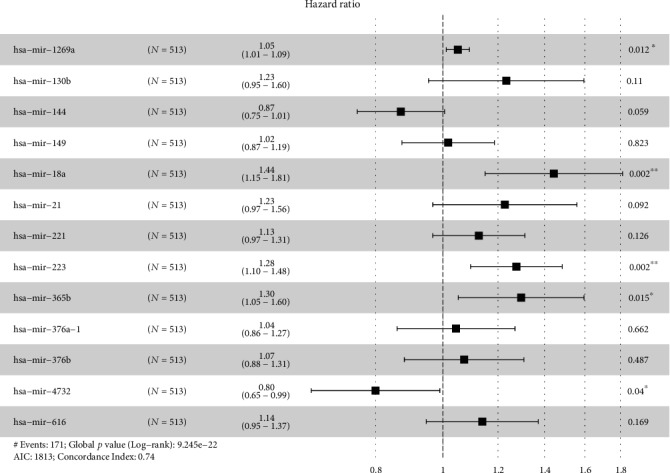
Multivariable Cox regression analysis of miRNA selection.

A total of 173 differentially expressed miRNAs were screened out. Among these miRNAs, 17 prognostic miRNAs were retained by the LASSO method. To build the optimal predictive signature, these potential miRNAs were further selected with a multivariate Cox regression analysis. Finally, 13 miRNAs were identified (Figure 1, Table 1), and a miRNA signature was constructed according to the expression levels of these miRNAs weighted by their relative coefficient derived from multivariate Cox regression. As a result, the prognostic risk score (miRNA signature) was determined for each of the 504 patients.

### 3.2. Nomogram Construction

**Figure 2:**
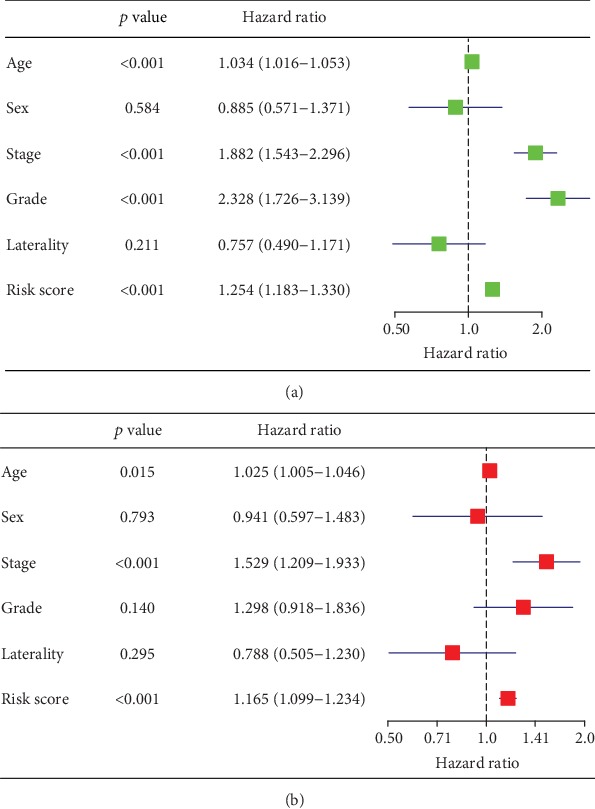
Univariate analyses (a) and multivariate analyses (b) to identify independent prognostic factors related to overall survival of patients with clear cell renal cell carcinoma in the development cohort.

**Figure 3:**
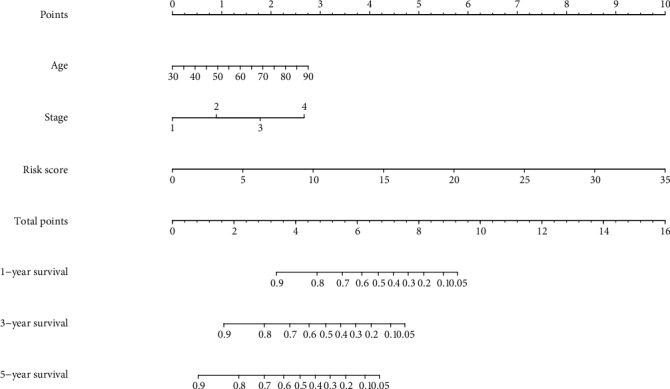
Nomogram for predicting 1-, 3-, and 5-year overall survival (OS) of patients with clear cell renal cell carcinoma.

**Figure 4:**
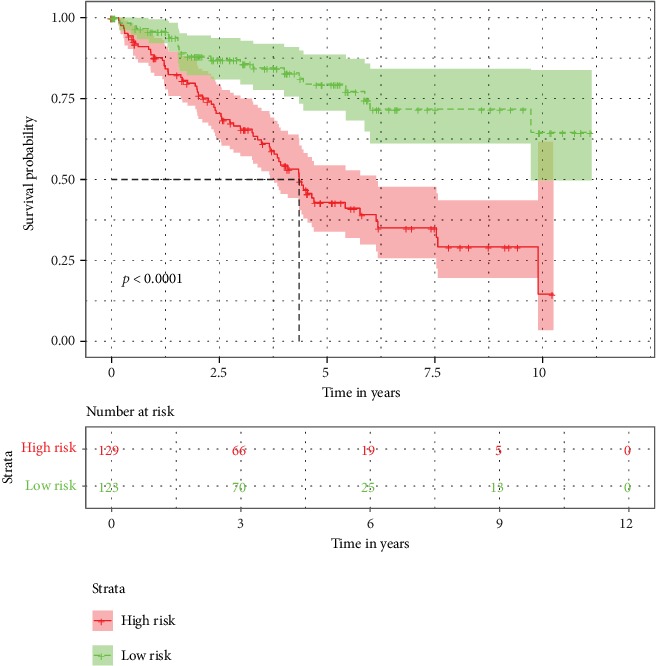
Kaplan-Meier survival curves of high-risk and low-risk patients grouped according to median risk score.

**Figure 5:**
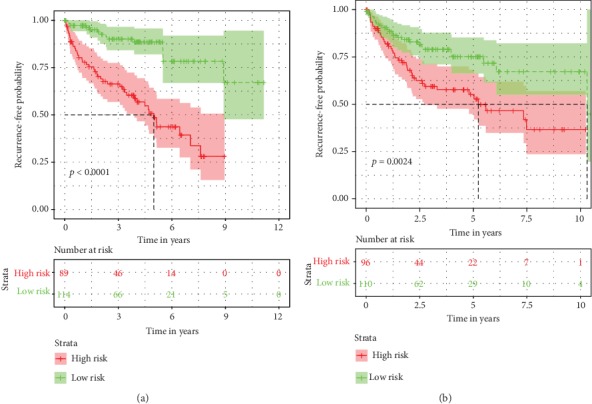
Kaplan-Meier plots of recurrence-free survival (RFS) based on high-risk and low-risk patients in with clear cell renal cell carcinoma.

The demographic and clinical characteristics of the development and validation cohorts are shown in Table 2. We used the univariate and multivariate Cox regression analysis to select independent predictors for patients with ccRCC in the development cohort. Risk score, age, grade, and AJCC stage were found to be related to survival in the development cohort (Figure 2(a)). A multivariate adjustment for other factors indicated that the risk score, age, and AJCC stage are independent prognostic factors (*P* < 0.005) in the development cohort (Figure 2(b)). A prognostic nomogram derived from these risk variables was established enabling the determination of an individual patient's score of each variable and the estimation of the probability of survival (Figure 3). Furthermore, in the validation cohort, all patients were classified into the high-risk group and low-risk group. Kaplan-Meier plots, tested by log-rank method, were used to establish the potential relationship between patients' survival and high-risk and low-risk groups. The Kaplan-Meier survival curve revealed that median survival time of the high-risk group is associated with shorter survival time than low-risk group with *P* value of <0.0001, as showed by the log-rank test (Figure 4). Moreover, the reproducibility of the miRNA signature in predicting the RFS of ccRCC patients in two cohorts was also determined. Patients were divided into a high-risk group and a low-risk group as previously determined. It was revealed by the log-rank test that the survival analyses of patients with low-risk group had higher RFS than those with high-risk group in the development cohort (Figure 5(a), *P* < 0.0001) and in the validation cohort (Figure 5(b), *P* = 0.0024).

### 3.3. Calibration and Validation of the Nomogram

**Figure 6:**
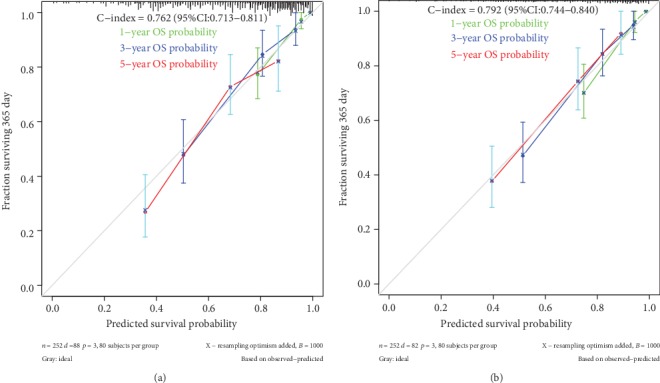
Calibration curves for predicting 1-, 3- and 5-year overall survival (OS) in the development (a) and validation cohorts (b). Bootstrap-predicted survival is plotted on the x-axis, and actual outcome is located on the y-axis. Vertical bars indicate 95% CIs derived from Kaplan-Meier analysis. Gray lines along the 1-slope diagonal line through the origin point denote an excellent calibration model.

We validated the performance of the nomogram in both cohorts. The nomogram exhibited favorable accuracy for OS prediction with a C-index of 0.792 (95% CI: 0.744-0.840). The C-index of the AJCC stages and miRNA signature alone for predicting survival in the development cohort was 0.731 (95% CI: 0.675-0.787), 0.778 (95% CI: 0.731-0.825), respectively, which were significantly lower than the nomogram. As for the validation cohort, the nomogram to predict OS also yielded favorable accuracy with a C-index of 0.762 (95% CI: 0.713-0.811). The C-index of the AJCC stages and miRNA signature alone for predicting survival was also lower than the nomogram at 0.717 (95% CI: 0.663-0.771), 0.685 (95% CI: 0.624-0.746), respectively. The calibration curves for the probabilities of 1-, 3-, and 5-year survivals indicated an excellent agreement between the nomogram prediction and observed outcomes in both the development and validation cohorts (Figure 6). These results indicate that the nomogram is better at predicting survival than the AJCC stage system and miRNA signature alone.

### 3.4. Decision Curve Analysis

**Figure 7:**
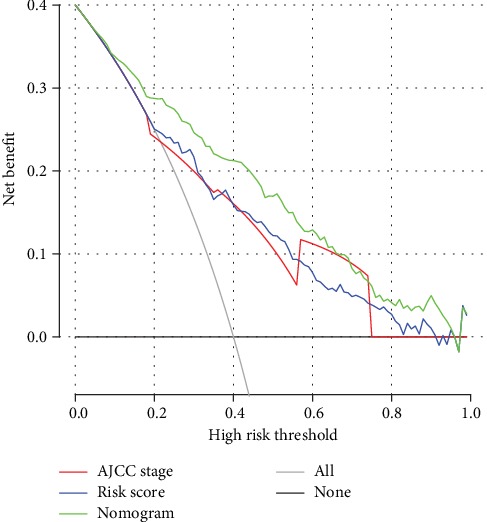
Decision curve analysis of nomogram and AJCC staging system and miRNA signature in terms of overall survival in the entire cohort. The x-axis indicates the threshold probability, and the y-axis demonstrates the net benefit. Abbreviations: AJCC staging system: American Joint Committee on Cancer staging system.

In DCA, the established nomogram presented a higher net benefit together with a broader range of threshold probability than the AJCC staging system and the miRNA signature (Figure 7), which demonstrates that our nomogram showed powerful predictive ability for survival.

## 4. Discussion

CcRCC is one of the most prevalent kidney cancers with high mortality and morbidity rates [20]. Nevertheless, current models for predicting patient survival only utilize conventional clinical parameters. For this reason, effective identification of other predictive variables is clinically challenging. The development of ccRCC is closely associated with the activation of proto-oncogenes, as well as the inactivation of tumor suppressor genes controlled by numerous miRNAs [21]. However, a single miRNA does not play a complete role in determining the prognosis of ccRCC.

Researches have confirmed that miRNA is closely associated with the occurrence of many cancers, and numerous miRNA have been used as novel biomarkers in cancers prognosis [22, 23]. Various miRNAs have been revealed to play an important role in tumor carcinogenesis. A study reported that three independent prognostic miRNAs (hsa-mir-144, hsa-mir-21, and hsa-mir-155) participating in the competing endogenous RNA network in ccRCC [24]. A study based on TCGA database identified four miRNAs, miR-149-5p, miRNA-21-5p, miRNA-9-5p, and miRNA-30b-5p, as independent prognostic indicators in patients with ccRCC. These miRNA target genes were mainly related to many pathways associated with tumor [25]. However, it failed to build a nomogram and compared with the presently used AJCC staging system. Nomograms provide individual predicts of future clinical outcomes by combining the effects of various variables associated with these events. The nomogram has proven to be a reliable tool for predicting the clinical survival of many types of cancers [26–30]. In this study, we screened 13 miRNAs, and a nomogram was then built based on the independent prognostic variables associated with patient survival. The nomogram exhibited favorable accuracy for OS prediction in both development and validation cohorts. The C-indexes of the AJCC stages and miRNA signature alone for predicting survival were also lower than the nomogram in both cohorts. The reproducibility of the miRNA signature in predicting the RFS of ccRCC patients in two cohorts was also determined. It is generally accepted that a C-index of >0.75 exhibits clearly useful discrimination [31]. These findings indicate that this novel prognostic model exhibits perfect performance.

In current clinical practice, the AJCC staging system has long been broadly used for prognostic evaluation of renal cancer [32]. However, many studies have suggested that this method has gradually lost its advantage in predicting patient survival time since other vital variables are neglected [14, 33]. A recent study established two prognostic nomograms that incorporated the log odds of positive lymph nodes for patients with RCC which found to be superior to the AJCC staging system in predicting cancer-specific survival and OS [34]. In our study, we corroborated this finding using a miRNA signature-based nomogram. However, perfect predictive ability does not indicate superior practicality in clinical practice [35]. Therefore, we used DCA to demonstrate that the novel nomogram has better clinical validity and applicability.

Although the novel nomogram demonstrated favorable accuracy, several limitations should be noted. Firstly, this nomogram was built based on the TCGA database, and external validation from other databases is needed. Secondly, several potential predictive factors were still not identified, including tumor size, chemotherapy, and radiotherapy, as they were unavailable in most patients.

## 5. Conclusion

In summary, miRNA signature is an independent prognostic factor for patients with ccRCC. The novel nomogram based on a miRNA signature is a powerful model for predicting the OS of patients with ccRCC compared to AJCC staging system, thus, improving clinical decision-making.

## Figures and Tables

**Table 1:** Prediction microRNAs and corresponding parameters in clear cell renal cell carcinoma.

microRNA	*β*	HR	HR 95% CI	*P* value
hsa-mir-1269a	0.04937	1.05061	1.0111-1.0917	0.01162
hsa-mir-130b	0.20987	1.23352	0.9537-1.5954	0.10983
hsa-mir-144	-0.13927	0.86999	0.7529-1.0053	0.05904
hsa-mir-149	0.01749	1.01764	0.8731-1.1861	0.82294
hsa-mir-18a	0.36738	1.44395	1.1497-1.8135	0.00158
hsa-mir-21	0.20522	1.2278	0.9671-1.5587	0.09191
hsa-mir-221	0.11894	1.12631	0.967-1.3119	0.12636
hsa-mir-223	0.24347	1.27567	1.096-1.4848	0.00167
hsa-mir-365b	0.25902	1.29566	1.052-1.5958	0.01482
hsa-mir-376a-1	0.0435	1.04446	0.8591-1.2697	0.66246
hsa-mir-376b	0.0702	1.07272	0.8799-1.3077	0.48733
hsa-mir-4732	-0.22316	0.79999	0.6466-0.9898	0.03995
hsa-mir-616	0.13038	1.13926	0.9463-1.3716	0.16851

Note: *β* indicates the regression coefficient; HR: hazard ratio; CI: confidence interval.

**Table 2:** Baseline demographic and clinical characteristics of the included patients with clear cell renal cell carcinoma.

	Development cohort (*N* = 252)	Validation cohort (*N* = 252)	*P* value
Age (mean ± SD)	60.90 ± 12.17	60.19 ± 12.00	0.5099
Sex (*N*, %)			0.02493
Male	152 (60.32)	176 (69.84)	
Female	100 (39.68)	76 (30.16)	
AJCC stage (*N*, %)			0.7451
I	123 (48.81)	125 (49.60)	
II	23 (9.13)	29 (11.51)	
III	65 (25.79)	57 (22.62)	
IV	41 (16.27)	41 (16.27)	
Grade (*N*, %)			0.1368
I	3 (1.19)	10 (3.97)	
II	102 (40.48)	113 (44.84)	
III	107 (42.46)	95 (37.70)	
IV	40 (15.87)	34 (13.49)	
Laterality (*N*, %)			1
Left	117 (46.43)	117 (46.43)	
Right	135 (53.57)	135 (53.57)	
Risk score (mean ± SD)	1.67 ± 2.73	1.62 ± 2.31	0.8244
Survival months (mean ± SD)	44.36 ± 31.96	44.47 ± 32.83	0.9696
Survival status (*N*, %)			0.57161
Dead	82 (32.54)	88 (34.92)	
Alive	170 (67.46)	164 (65.08)	

SD: Standard Deviation; AJCC: American Joint Committee on Cancer.

## Data Availability

The raw data of this study are derived from the TCGA data portal (http://tcga.cancer.gov), which is a publicly available database.
